# ASHP Survey of Health-System Specialty Pharmacy Practice: Clinical Services—2024

**DOI:** 10.1093/ajhp/zxag130

**Published:** 2026-04-25

**Authors:** Autumn D Zuckerman, Gabrielle Pierce, Erica Diamantides, Jennifer Loucks, Scott Canfield, Kimhouy Tong, Josh DeClercq

**Affiliations:** Vanderbilt Specialty Pharmacy, Vanderbilt Health, Nashville, TN, USA; American Society of Health-System Pharmacists, Bethesda, MD, USA; Tufts Medicine, Boston, MA, USA; The University of Kansas Health System, Kansas City, KS, USA; Johns Hopkins Care at Home, Baltimore, MD, USA; Outpatient Pharmacy Services, Yale New Haven Health, Hamden, CT, USA; Department of Biostatistics, Vanderbilt University Medical Center, Nashville, TN, USA

**Keywords:** clinical services, pharmacy practice model, specialty medication, specialty pharmacy, survey

## Abstract

**Purpose:**

Results of the second ASHP Survey of Health-System Specialty Pharmacy Practice: Clinical Services are presented.

**Methods:**

The 2022 clinical services survey questionnaire was reviewed by a panel of specialty pharmacy experts and reduced to 64 questions. The convenience survey sample of 298 unique health-system specialty pharmacies (HSSPs) was compiled from ASHP member lists, the presence of specialty pharmacies indicated in previous ASHP surveys, and outreach to ASHP member organizational leaders. The survey was hosted using Qualtrics.

**Results:**

The response rate was 23.8% (71/298). Most respondents reported dispensing less than 45,000 specialty prescriptions annually, have offered specialty pharmacy services for more than 7 years, and are part of an academic medical center. Over half of HSSPs (61%) reported being able to fill less than half of the specialty medication prescriptions at their health system, most commonly due to payer network restrictions. HSSPs provide many access-related services to all patients of the health system regardless of their ability to fill the specialty prescription, and most respondents (77%) reported offering services beyond basic specialty medication prescription fulfillment. Most HSSPs reported tracking patient response to therapy using disease state–specific outcomes and documenting all interventions in the electronic health record. HSSPs heavily contribute to the field of specialty pharmacy through research and education.

**Conclusion:**

The HSSP model is a continually maturing, integrated advanced practice model focused on providing patient-centric care to all health-system patients regardless of network status, yet access to fill prescriptions written at the health system remains limited.

Key Points

- Health-system specialty pharmacies (HSSPs) continue to advance the practice model by servicing new clinical areas, expanding technology and collaborative practice agreement uses, and creating new service lines to address the evolving needs of specialty patients and medications.
- Using the electronic health record, HSSPs couple clinical outcome data with operational data to create a transparent view of the patient journey that informs tailored interventions within each step of the journey and optimizes medication access and outcomes.
- HSSPs drive innovation and practice advancement through research, publications, and interdisciplinary healthcare education.

The 2024 ASHP Survey of Health-System Specialty Pharmacy Practice: Clinical Services is the fourth survey of its kind and is the second iteration focusing specifically on clinical services provided by health-system specialty pharmacies (HSSPs).^1–3^ As hospitals and health systems continue to expand their specialty pharmacy services, the clinical services offered by these HSSPs evolve to optimize use of specialty medications and patient outcomes. The growth of rare disease and ultra-complex specialty medications necessitates a more intentional and patient-centered approach to specialty medication management than ever before.^4^ HSSPs represent an advanced, and often misunderstood, practice model that support patients prescribed specialty medications from medication selection through treatment monitoring and optimization. However, the differences between HSSP models and their capabilities are often highlighted as a challenge to understanding this model of specialty pharmacy.

This survey, building on 3 previous surveys on the HSSP practice model and clinical services, provides up-to-date insight into the integration and impact of HSSPs throughout the specialty patient journey.^1–3^ In the time since the previous survey focused on clinical services in 2022 was conducted, significant societal and regulatory changes have occurred, including the latter years of the COVID-19 pandemic and passage of the Inflation Reduction Act.^5^ As the specialty medication landscape continues to become more complex, with sustained growth in specialty medication healthcare expenditures and new approvals of specialty medications, these results provide an in-depth review of the current state of HSSPs’ clinical services.^6^

## Methods

### Questionnaire development

An expert advisory panel (Appendix A) comprising of thought leaders in HSSP from the Vanderbilt Health System Specialty Pharmacy Outcomes Research Consortium study group that developed the first clinical services survey and leaders within the ASHP Section of Specialty Pharmacy Practitioners was convened in October 2023 to review questions from the previous survey for clarity, responses, and relevance. The panel met 5 times between October and December 2023. Pilot testing with the expert panel occurred in February 2024. The questionnaire consisted of 64 questions. The previous survey, conducted in 2022, consisted of 119 questions; the 2024 questionnaire was shortened by removing areas of duplication, focusing on questions related to clinical practices, streamlining question formatting, and removing questions that did not contribute to meaningful results. This survey focused on 7 domains: (1) respondent demographics, (2) general capabilities, (3) pre-treatment, (4) treatment initiation, (5) treatment monitoring, (6) continuity of care services, and (7) education and training. The survey was not institutional review board approved, and the research was conducted according to the principles of the Declaration of Helsinki, seventh revision.^7^

### Survey sample

As noted in previous surveys, there is no common repository containing all United States–based HSSPs. Therefore, a convenience sample of leaders at HSSPs identified by ASHP was used to distribute the survey via email. This yielded contacts from 298 unique HSSPs. The first survey question screened respondents to ensure they worked within an HSSP, aligning with the population of interest for this survey.

### Data collection

Survey completion was done online using Qualtrics (Qualtrics, Provo, UT). HSSP leaders were contacted a maximum of 4 times during the survey period to request survey completion and for deadline reminders. The first contact was on May 17, 2024, and the last contact was on June 27, 2024. Nonresponders were sent reminder emails approximately every 2 weeks. In addition, the following information pushes were conducted: an ASHP email Newslink summary; invitation messaging to leaders at large specialty pharmacy vendors, collaborative networks, and group purchasing organizations requesting that they share the survey link with specialty pharmacy director contacts; and direct emails to current and former ASHP Section of Specialty Pharmacy Practitioners volunteer leaders and advisory group members. Data collection was closed on June 28, 2024.

### Data analysis

Duplicate responses (n = 13) from health systems were eliminated after evaluating emails of respondents. Survey completion progress was calculated based on the number of questions answered divided by the total number of questions, and the most complete response between duplicates was used. All completed and in-progress responses were used. Data were output from Qualtrics into an SPSS-readable file. Descriptive statistics, including medians, interquartile ranges (IQRs), frequencies, and percentages, were used to summarize the survey results. Data visualization was performed using graphs. All data analyses and graphs were generated using R (version 4.4.0; R Foundation for Statistical Computing, Vienna, Austria).

## Results

### Respondents

The response rate was 23.8% (71/298), similar to the 29% response rate from the 2022 survey. Respondent demographics are shown in Table 1. Most respondents were from mature programs, with 40% having offered specialty pharmacy services for 7 to 10 years and 31% being established for 11 or more years (therefore, 71% were established for more than 7 years). Similar to 2022 survey data, approximately two-thirds of respondents (67%) were affiliated with an academic medical center. The number of prescriptions dispensed in the last year varied, with 44% of HSSPs dispensing less than 30,000 prescriptions and 33% dispensing more than 60,000 prescriptions. The proportion of respondents reporting they are able to dispense less than half of the specialty prescriptions written at their institution increased to 61% in the current survey, compared to 48% in 2022. A new question on the 2024 survey provided insight into where restrictions are most prevalent, with a median of 36% of prescriptions being ineligible to be filled due to payer lockouts (Figure 1).

**Figure 1.**
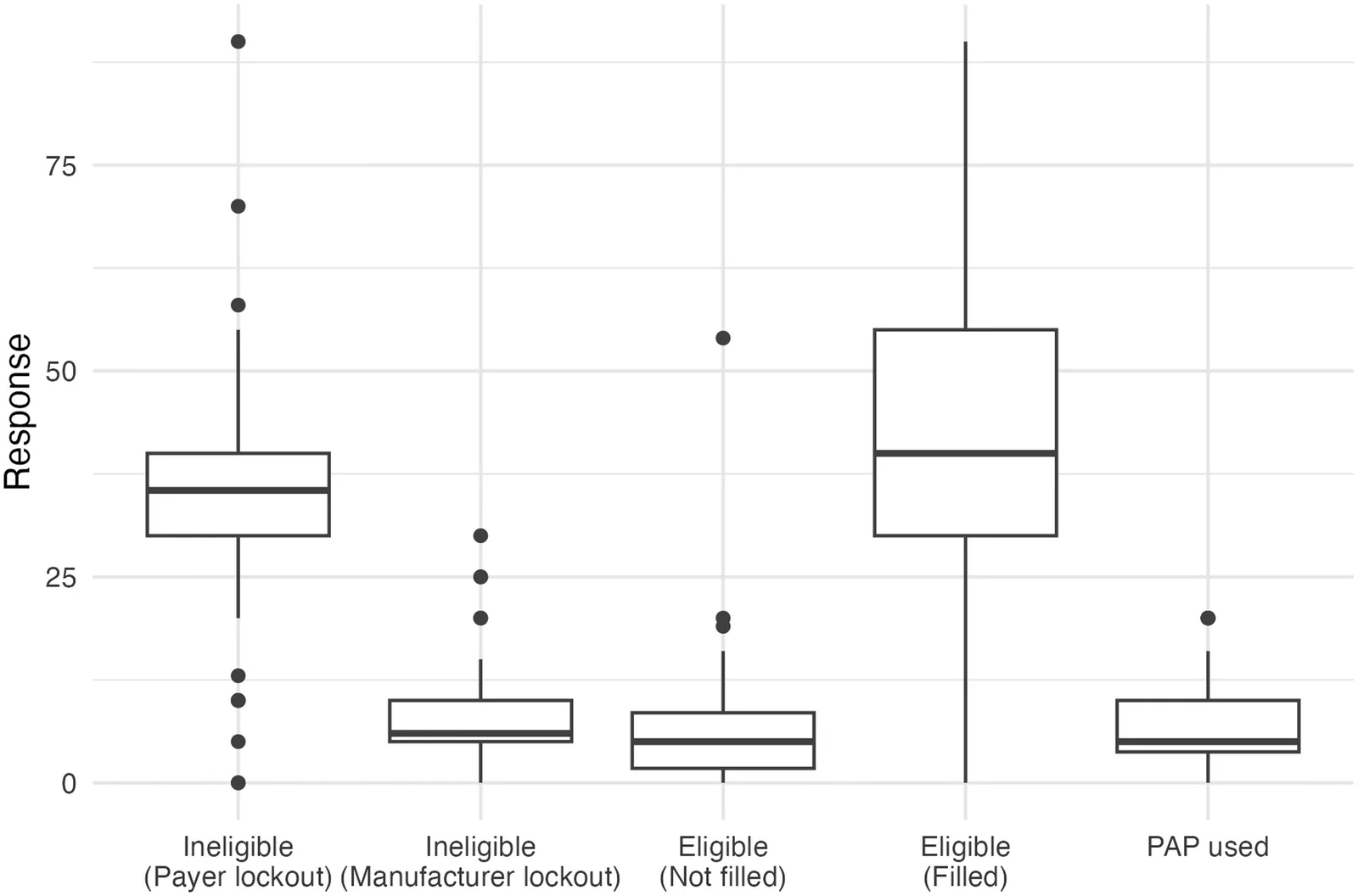
Of the specialty medication prescriptions generated at responding health-system specialty pharmacies (HSSPs), respondents reported a median of 40% were eligible and filled at the HSSP, with 36% estimated to be ineligible due to payer lockout, 7% ineligible due to manufacturer lockout, 5% filled through manufacturer patient assistance programs (PAPs), and 5% eligible to be filled at the HSSP but not filled.

**Table 1.** Health-System Specialty Pharmacy Characteristics by Survey Year^a^

Survey question	2022 (N = 127)	2024 (N = 71)
**How long has your health system offered specialty pharmacy services? (select one)**		
Less than 1 year	4 (3)	0 (0)
1-3 years	23 (18)	3 (4)
4-6 years	37 (29)	17 (24)
7 or more years	61 (48)	NA
7-10 years	NA	28 (40)
11 or more years	NA	22 (31)
My health system does not offer specialty pharmacy services	2 (2)	0 (0)
Missing	0	1
**Does your health system include an academic medical center (eg, organizationally and administratively integrated with a medical school)?**		
No	44 (36)	21 (33)
Yes	78 (64)	43 (67)
Missing	5	7
**In the last fiscal year, how many specialty pharmacy prescriptions were filled by your specialty pharmacy operation? Provide your aggregated information regardless of whether your health system has a dedicated specialty pharmacy or dispenses specialty prescriptions from multiple health-system pharmacies. (select one)**		
Less than 7,500	NA	7 (11)
7,501-15,000	NA	8 (13)
<15,000	46 (40)	NA
15,001-30,000	24 (21)	13 (20)
30,001-45,000	17 (15)	9 (14)
>45,000	29 (25)	27 (42)
45,001-60,000	NA	6 (9)
60,001-75,000	NA	13 (20)
>75,000	NA	8 (13)
Missing	11	7
**What percentage of all specialty pharmacy prescriptions originating from your health-system providers are filled by your specialty pharmacy? (select one)**		
Less than 25%	6 (5)	8 (13)
25%-50%	49 (43)	31 (48)
51%-75%	36 (32)	18 (28)
Greater than 75%	9 (7)	7 (11)
Unsure	13 (12)	0 (0)
Missing	14	7
**Select the model that best describes your health-system specialty pharmacy: (select one)**		
Generalist model: most specialty pharmacists cover all disease states	34 (30)	13 (25)
Hybrid model: roughly even mix of generalists and specialists	33 (29)	18 (35)
Specialist model: most specialty pharmacists are dedicated to specific disease states	48 (42)	21 (40)
Missing	12	19

Abbreviation: NA, not asked.

^a^All data are No. (%) of survey respondents. Missing data was not used in calculating percentages.

Most respondents continued to have primarily a specialist (40%) or hybrid (35%) model, wherein outpatient pharmacists are dedicated to a specific clinical area or a mix of generalists and specialists, respectively. Dedicating full HSSP support (eg, prescription fulfillment, prior authorization completion, monitoring) was much more common than partial HSSP support (eg, prior authorization [PA] completion and initial counseling but no monitoring), with respondents reporting a median of 15 (IQR, 10-30) versus 2 (IQR, 0-4) specialty areas in their health system with full and partial support, respectively. Both pharmacists and pharmacy technicians are commonly dedicated to a specific clinical area, with 46% and 40% of respondents reporting dedicated pharmacist and technician personnel, respectively, for more than 75% of specialty areas serviced (Figure 2).

**Figure 2.**
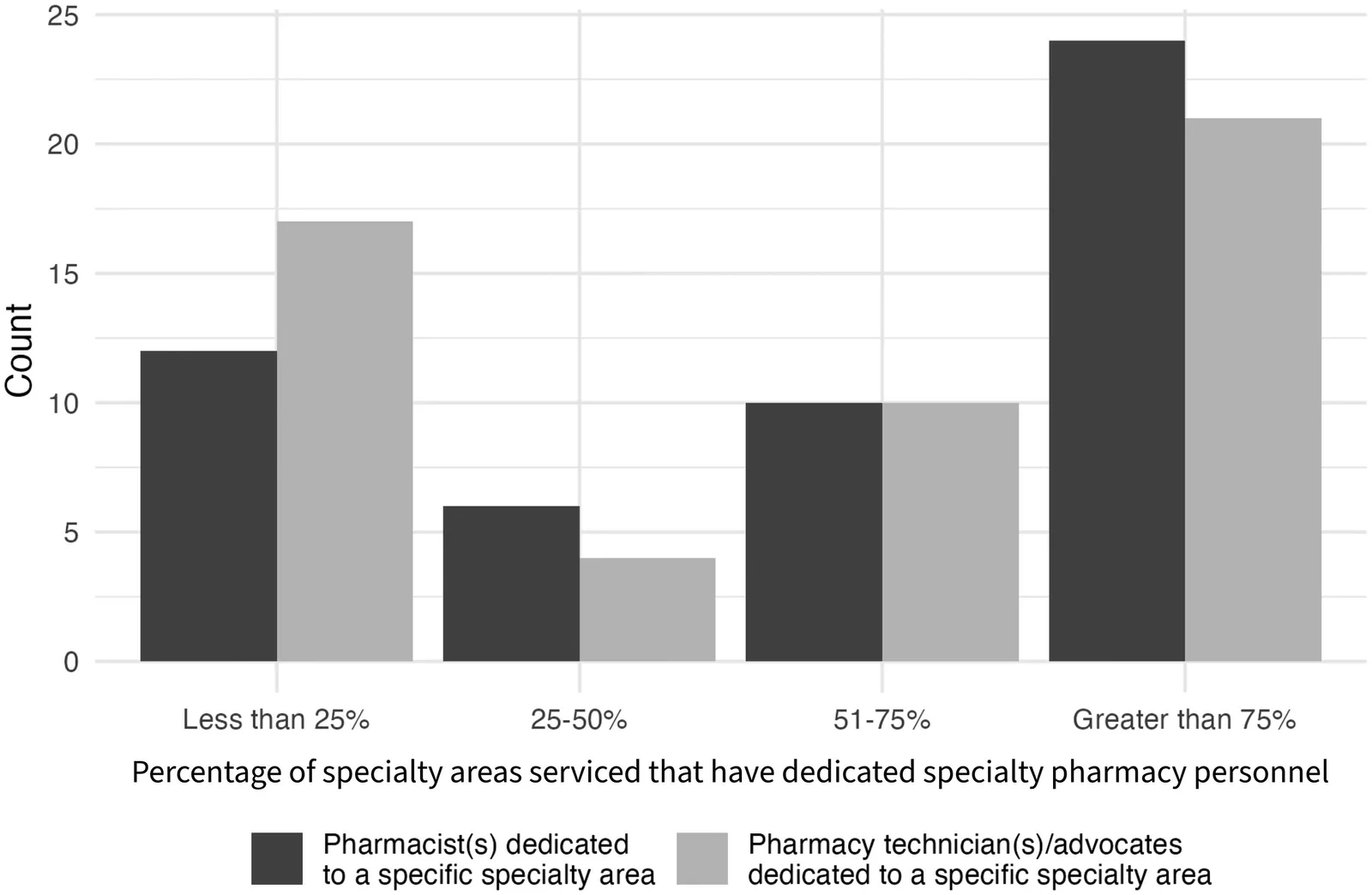
Most respondents dedicated pharmacists and pharmacy technicians to over 50% of the specialty clinical areas serviced by the health-system specialty pharmacy.

Clinical areas serviced remained similar to those reported in the 2022 survey, with oncology (98%), hematology (92%), rheumatology (92%), multiple sclerosis (92%), hepatitis C virus (90%), and adult gastrointestinal (89%) being the most common areas. Notable growth was seen in the following areas in comparison to 2022: pulmonary (81% in 2024, 60% in 2022), pediatric endocrinology (50% in 2024, 37% in 2022), and sickle cell disease (37% in 2024, 25% in 2022). Newly added categories, including allergy/immunology (71%), nephrology (39%), and hemophilia/bleeding disorders (29%), provided insight into these growing areas. The clinical area “rare diseases” was noted by 3 respondents in the “other” category (Figure 3).

**Figure 3.**
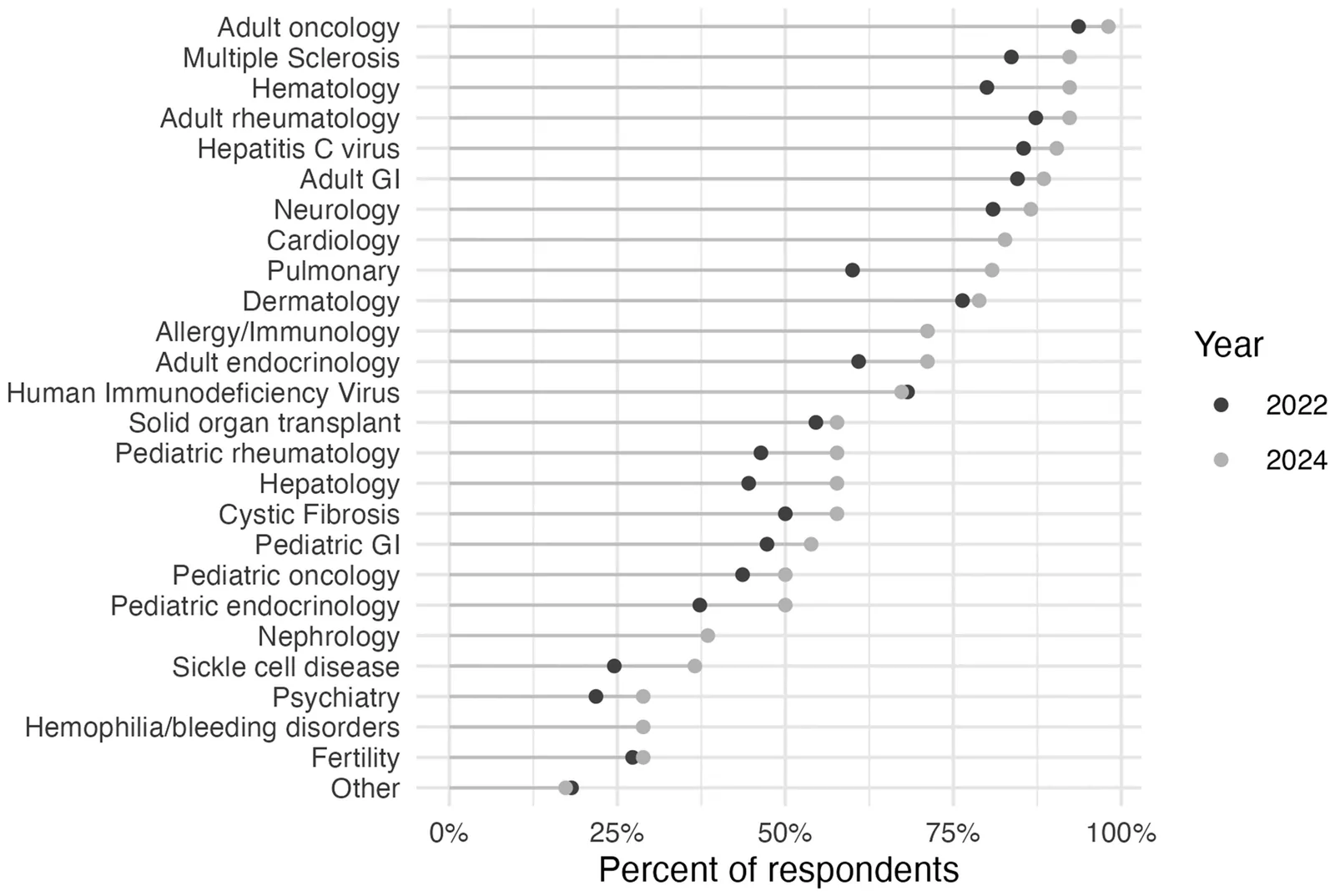
Clinical areas reported to be serviced by health-system specialty pharmacies (HSSPs) in 2022 (dark gray) and 2024 (light gray). GI indicates gastrointestinal.

### General capabilities

Ten questions evaluated HSSP general capabilities. HSSPs continue to provide a plethora of services to patients seen at the health system regardless of their ability to fill the specialty prescription. The most commonly offered services were PA completion assistance (82%), PA denial assistance (72%), financial assistance resources (58%), and free drug program management (63%). HSSPs continue to report involvement in specialty medication selection, with 58% of respondents reportedly providing specialty medication selection and/or recommendation for all patients referred to the HSSP and 26% providing these services for those receiving medication through the HSSP only. For patients receiving medication through the HSSP only, the most common services provided were related to clinical evaluation and monitoring and care coordination, including refill reminders (92%), adherence monitoring (90%), and individualized patient care plans (80%) (Figure 4). Risk evaluation and mitigation strategies (REMS) management services remained more commonly provided only to patients receiving medication through the HSSP (72% in 2024, 67% in 2022).

**Figure 4.**
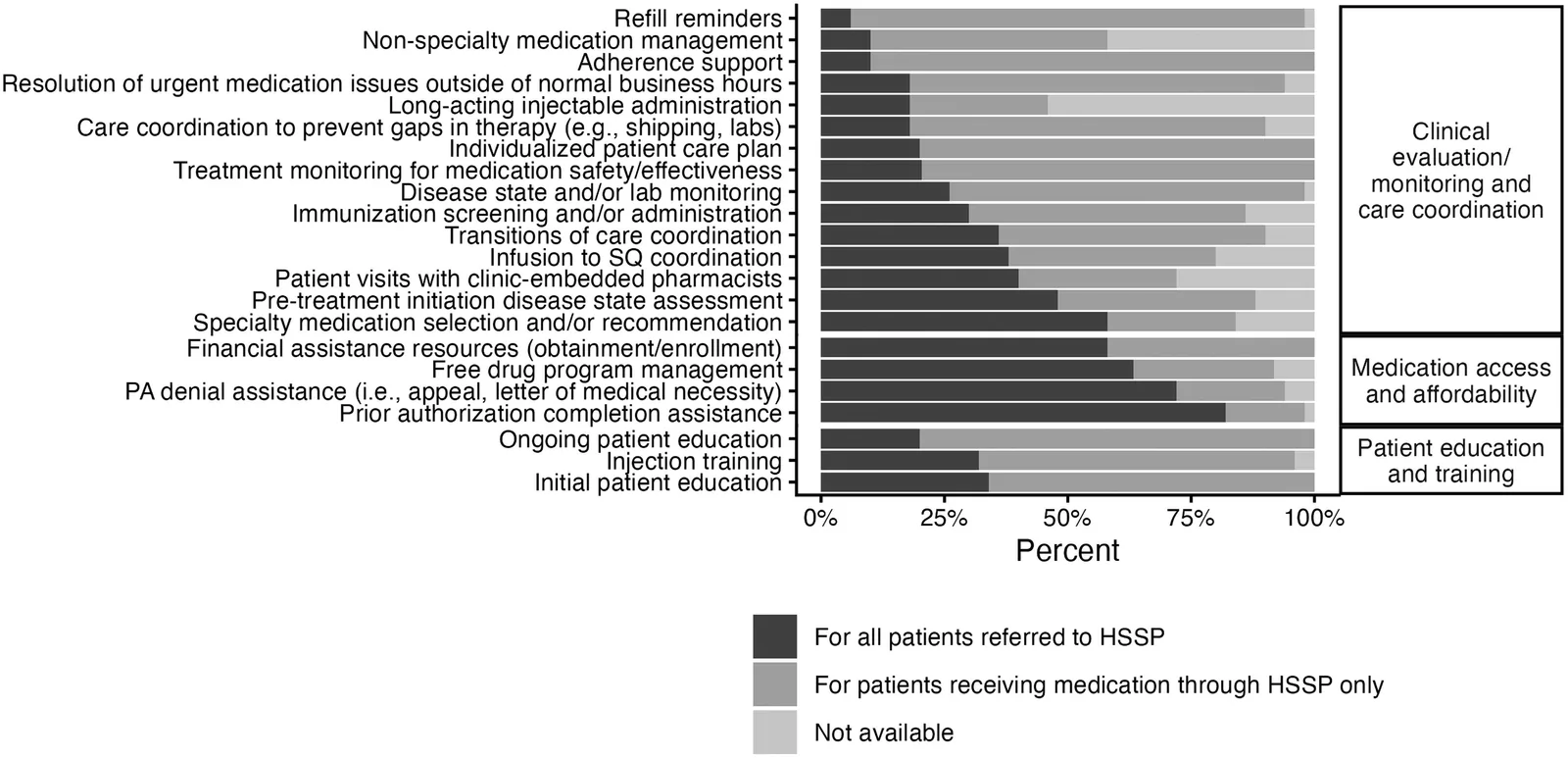
Health-system specialty pharmacies (HSSPs) provide many services to all patients referred regardless of their ability to fill the prescription (dark gray) at the HSSP, primarily services related to medication access. Clinical services such as treatment monitoring and adherence support were more commonly provided to only patients filling with the HSSP (middle gray). Few services included in the survey were not provided (light gray). SQ indicates subcutaneous.

The use of collaborative practice agreements (CPAs) and/or collaborative drug therapy management (CDTM) remained relatively stable, with a slight decrease in the percentage of respondents reporting no CPA/CDTM use (44% in 2024, 47% in 2022) (Figure 5). In response to a new question added to the 2024 questionnaire, respondents shared that CPA/CDTM arrangements commonly include prescribing renewal medication (82%), ordering laboratory tests (70%), and prescribing ancillary medications related to specialty conditions (63%) (Figure 6). Over half (52%) reported prescribing initial medications under a CPA/CDTM. Billing for services provided by HSSPs is rare (6%).

**Figure 5.**
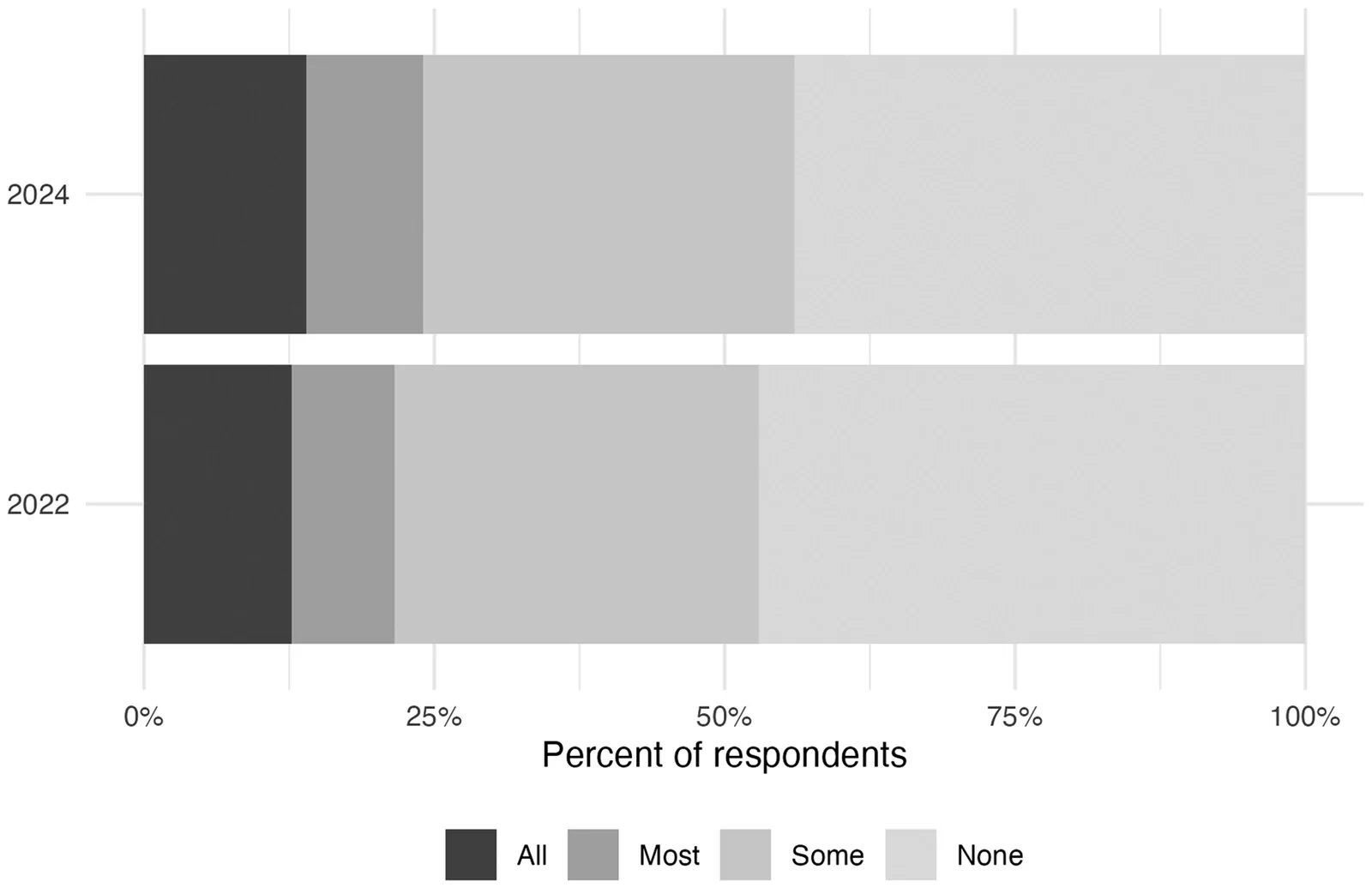
Results regarding use of collaborative practice agreements (CPAs) and/or collaborative drug therapy management (CDTM) in 2022 and 2024 are shown. In 2024, over half of health-system specialty pharmacies (66%) reported CPA/CDTM use in at least some clinical areas. Only 13% reported CPA/CDTM use in all clinical areas (dark gray), and 44% reported no CPA/CDTM use (light gray).

**Figure 6.**
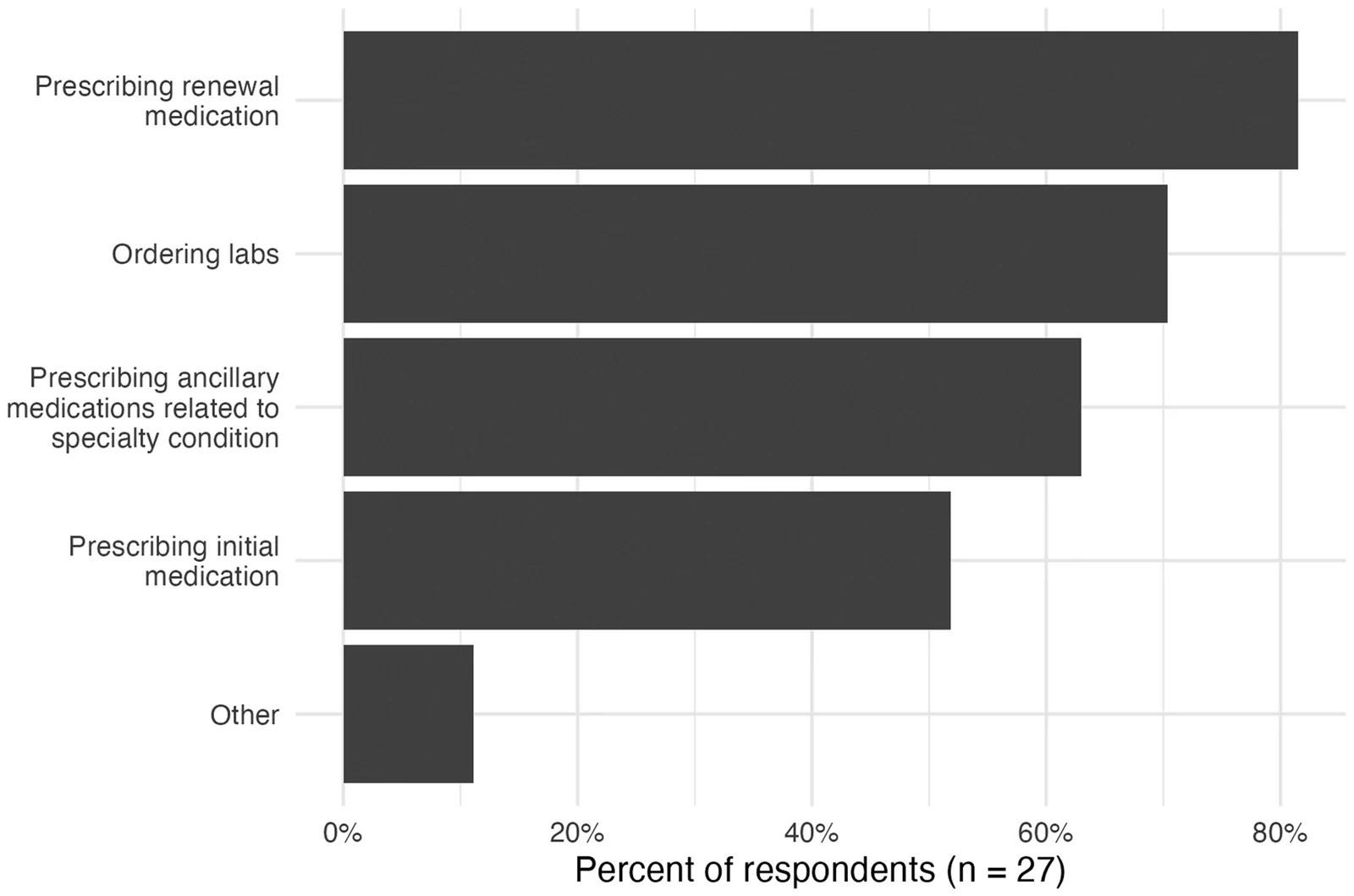
Services covered by collaborative practice agreements (CPAs) and/or collaborative drug therapy management (CDTM) were broad, most commonly encompassing prescribing renewal medications and ordering laboratory tests. Over half (55%) of CPA/CDTM arrangements covered prescribing of initial treatment.

Transparency of HSSP activities to providers using the shared electronic health record (EHR) remained strong, with more than 75% of respondents reporting that all surveyed activities were sometimes, frequently, or always documented and visible to providers. The top 4 activities documented in the EHR (combining “frequently” and “always” responses) were initial patient counseling notes (86%), patient concerns/preferences regarding treatment (87%), process and status of insurance navigation and financial assistance procurement (84%), and patient response to therapy (82%) (Figure 7).

**Figure 7.**
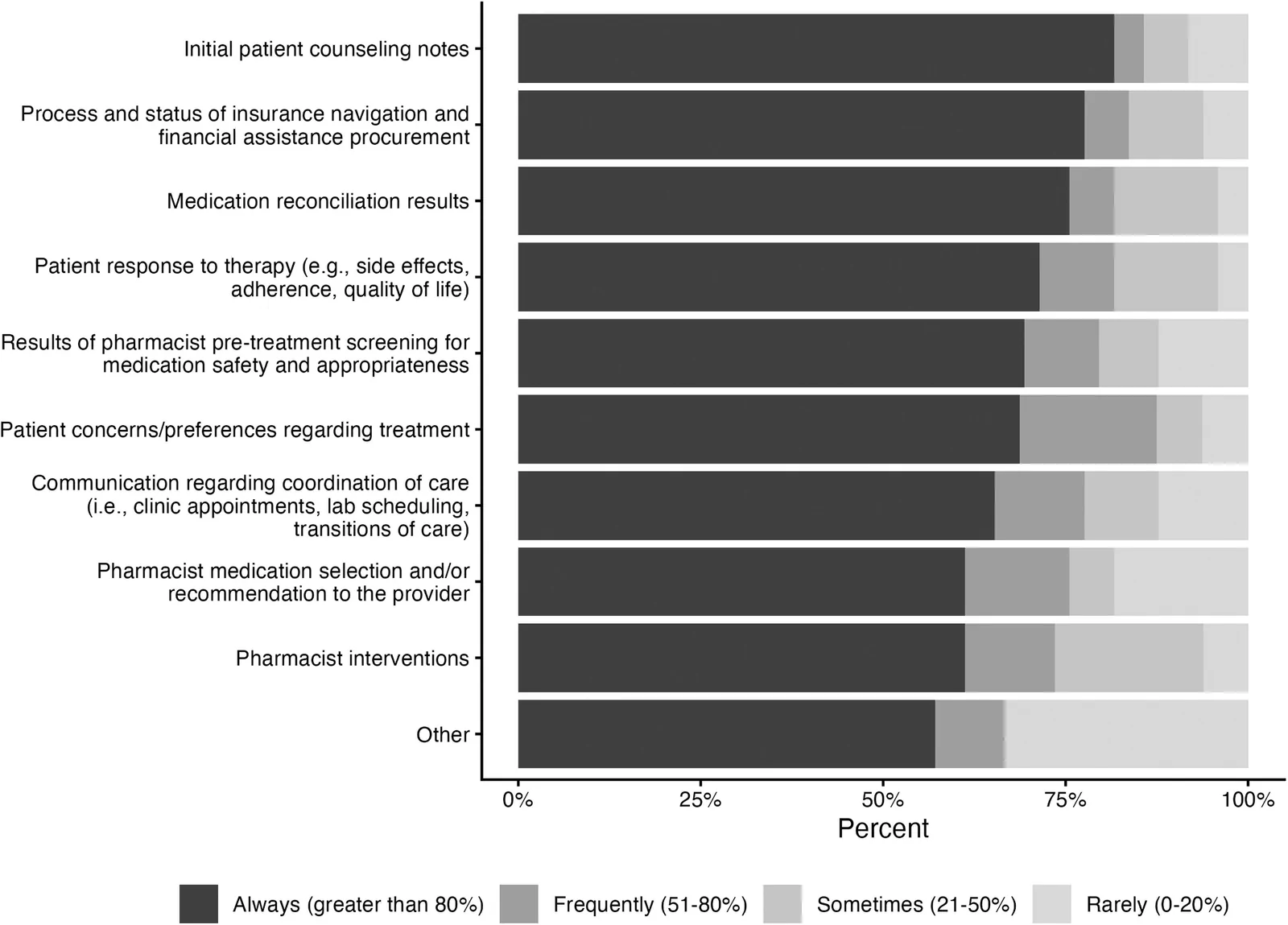
Transparency into the patient journey is a key differentiator of health-system specialty pharmacy. All actions surveyed were reported to be frequently or always documented in the electronic health record by over half of respondents.

Data collection and reporting continues to be a common requirement for HSSPs to demonstrate value to stakeholders. Most data are reported to health-system clinic providers and staff or leadership (Figure 8). The prevalence of reporting time to treatment has increased since 2022, with a 21 percentage point increase in reporting this metric to health-system leadership (83% in 2024, 62% in 2022), a 23 percentage point increase in reporting it to manufacturers (63% in 2024, 40% in 2022), and a 15 percentage point increase in reporting it to payers (61% in 2024, 46% in 2022). Overall, HSSPs most commonly report time to treatment, patient satisfaction, patient clinical outcomes, prescription volume, and adherence to demonstrate value to payers and manufacturers. Pharmacist interventions and insurance approval rates were new options in the 2024 survey and were commonly reported to health-system providers and leaders. Interventions were also communicated to manufacturers and payers somewhat often (Figure 8).

**Figure 8.**
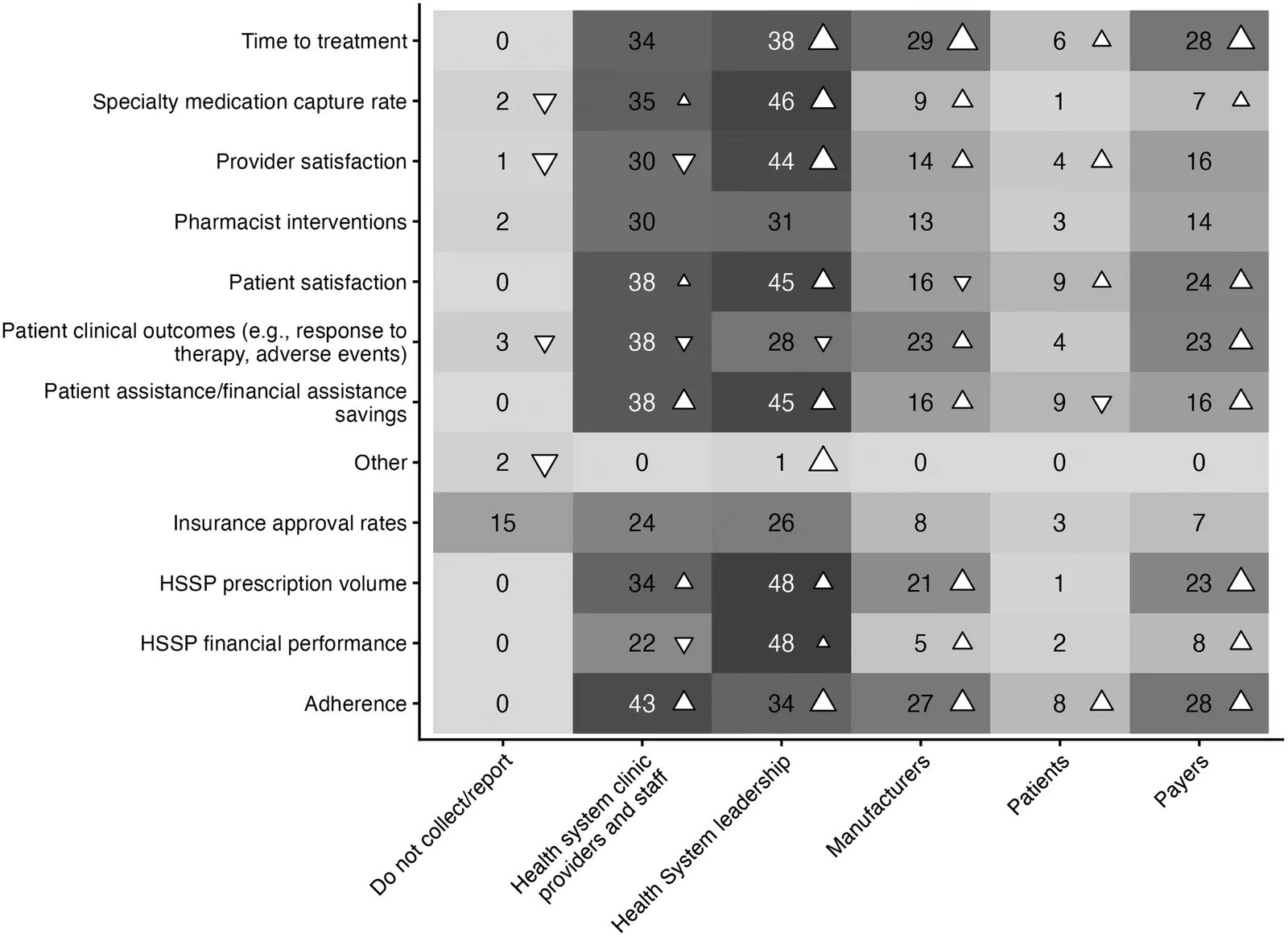
Health-system specialty pharmacies (HSSPs) use several data points to demonstrate value to stakeholders, most commonly internally within the HSSP. Metrics surveyed appear on the y-axis, and stakeholders to which data might be communicated are listed on the x-axis. The numbers embedded in cells represent raw counts of respondents out of a total of 52 respondents to this question. Darker cells indicate higher counts. The direction and size of the up- and down-pointing triangles indicate the direction and magnitude of percentage change between the 2022 and 2024 survey results.

### Pre-treatment

Survey participants were asked 6 questions regarding specialty pharmacist actions and interactions that occur once a decision to treat has been made but prior to treatment selection and/or initiation of therapy. The frequency at which providers seek pharmacy input to evaluate insurance coverage and treatment affordability remained relatively unchanged from the previous survey, with 49% reporting frequently or always in 2024 versus 46% in 2022.

Specialty pharmacies continued to report that pharmacists have a role in specialty medication selection. Almost all HSSPs reported that pharmacists frequently or always review provider medication selection and alert the provider with concerns. Over three-fourths (77%) reported collaboratively discussing treatment selection with providers, and many reported the pharmacist frequently or always made the therapy selection, either authorized by the provider (32%) or under a CPA/CDTM (23%) (Figure 9).

**Figure 9.**
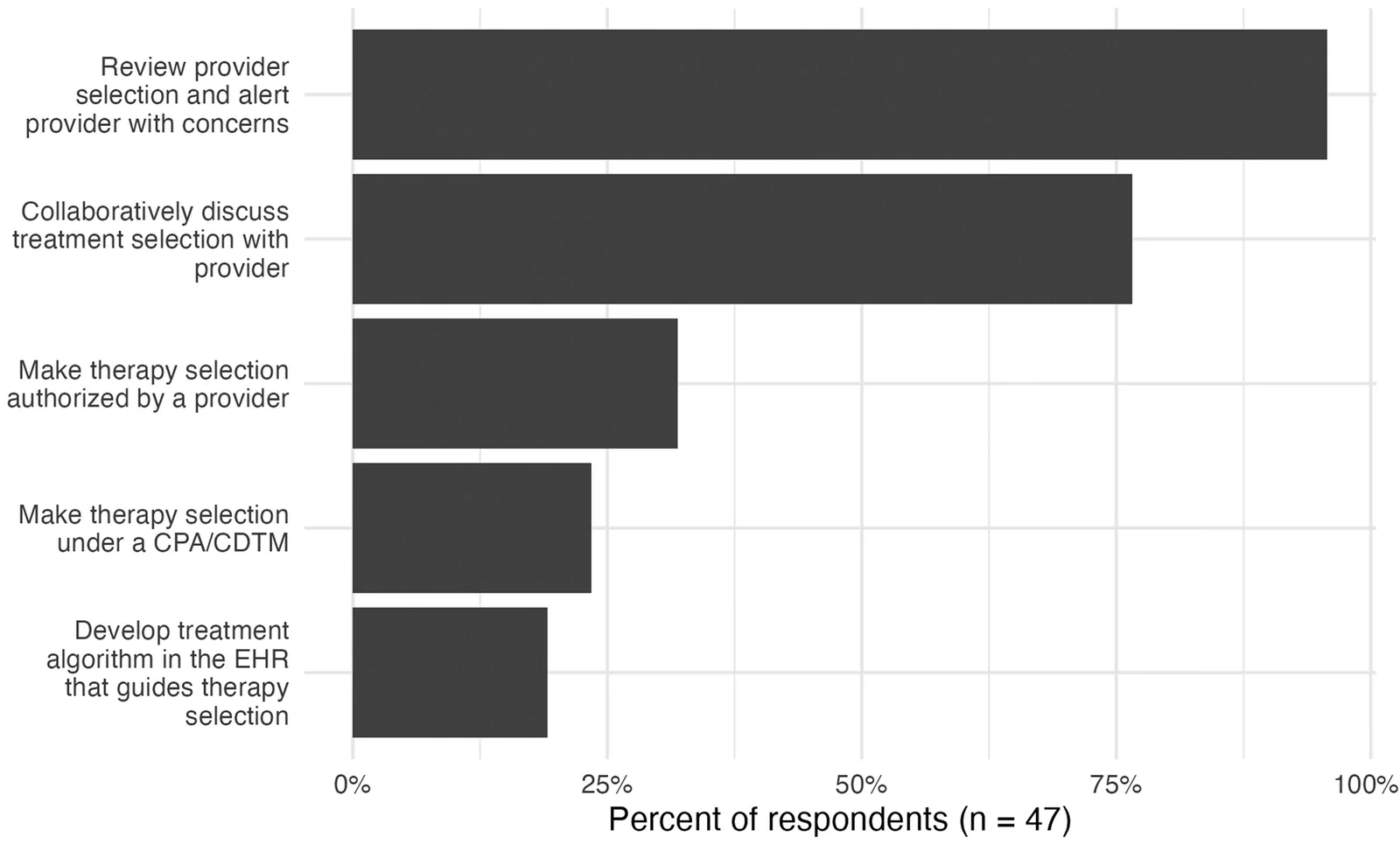
Health-system specialty pharmacy pharmacists reported an active role in specialty medication selection, as indicated by responses to the question “Which of the following are specialty pharmacists able to perform related to medication selection in most of the clinics serviced by the health system specialty pharmacy frequently or always (greater than 50% of the time)?” CPA indicates collaborative practice agreement; EHR, electronic health record; CDTM, collaborative drug therapy management.

In the 2024 survey, over 90% of respondents reported using the EHR for evaluation of pre-treatment safety parameters, baseline disease status, and comorbidities prior to starting treatment, which was comparable to 2022 results. If safety screening merits intervention prior to therapy initiation, a larger proportion of institutions reported pharmacists taking action through directly alerting the patient that additional pre-treatment workup is needed prior to starting therapy (34% in 2024 vs 3% in 2022) or through a CPA compared to the previous survey (15% in 2024 vs 5% in 2022). Fewer respondents reported pharmacists alerting the provider or clinic that necessary pre-treatment workup is required (4% in 2024 vs 41% in 2022).

The percentage of HSSPs reporting frequently or always providing patient counseling prior to medication selection (52%) increased from the previous survey’s 30% (Figure 10). Telephone communication remained the most commonly reported pre-treatment counseling method, with approximately 60% of respondents reporting using telephone for pre-treatment counseling frequently or always. Fewer respondents reported frequently or always providing pre-treatment counseling during a provider visit in person (23%) compared to the previous survey (44%). Reported rates of pre-treatment counseling in a separate clinic visit in person remained similar to the previous survey.

**Figure 10.**
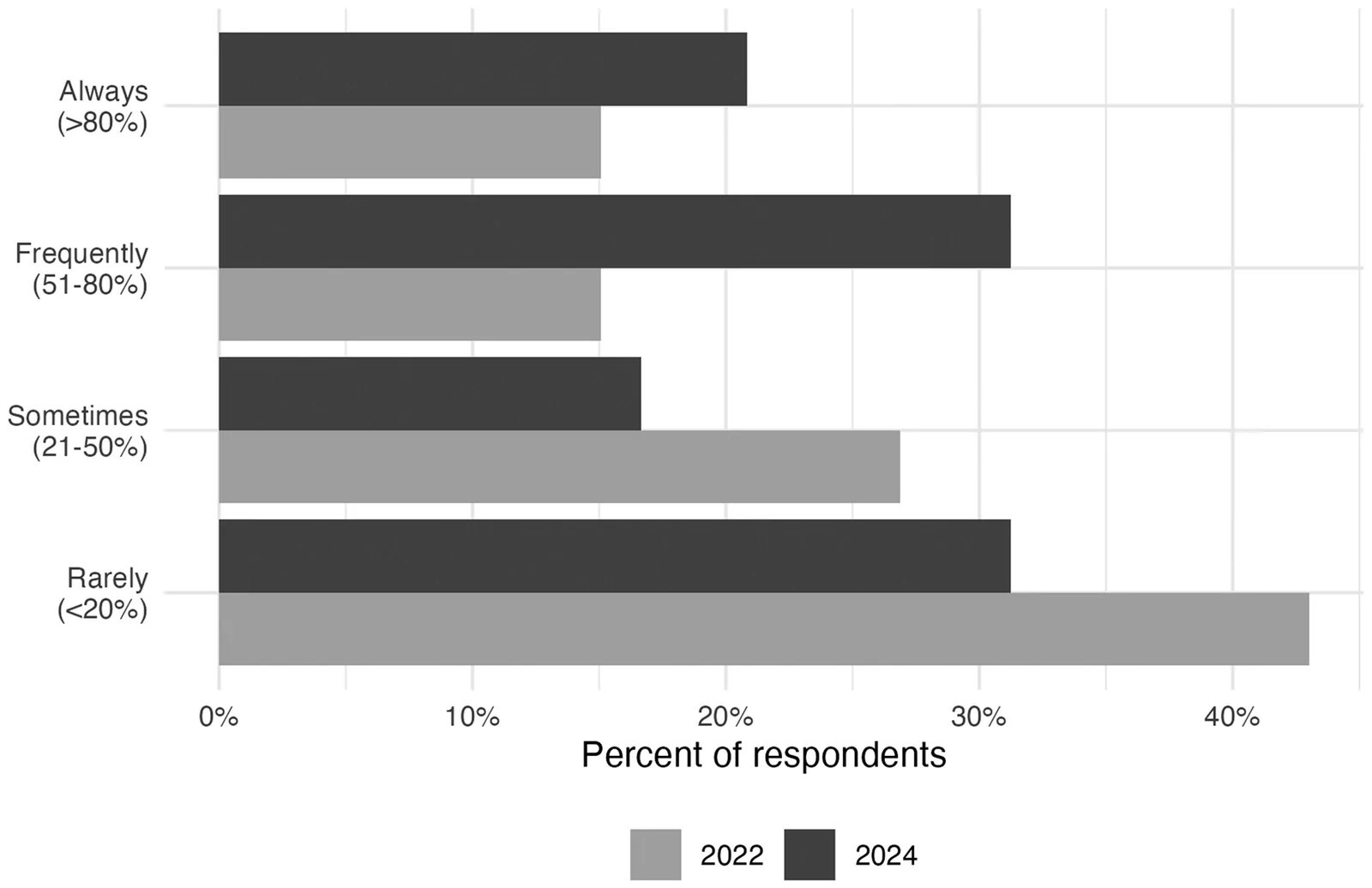
The frequency with which health-system specialty pharmacies reported that patients receive pre-treatment counseling prior to medication selection in the 2022 (light gray) and 2024 (dark gray) surveys.

### Treatment Initiation

Survey participants were asked 3 questions pertaining to treatment initiation activities (Table 2). Similar to pre-treatment counseling, telephone contact remained the most common method of injection counseling (67% of participants reported this occurred frequently or always) and initial medication counseling (80% frequently or always). In-person injection counseling was less commonly frequently or always performed in a separate clinic visit (10%) or during a provider visit (12%).

**Table 2.** Treatment Counseling Methods (N = 49 Responses)^a^

Survey question	Rarely (0%-20%)	Sometimes (21%-50%)	Frequently (51%-80%)	Always (>80%)	None selected
**Please select your estimate of the frequency with which pharmacists perform injection counseling through each of the following methods in most of your clinics: (select one for each method)**					
During a separate clinic visit in person	25 (51)	15 (31)	7 (14)	0	2 (4)
During provider visit in person	28 (57)	17 (35)	1 (2)	4 (8)	0
Via telemed video counseling	3 (6)	10 (20)	2 (4)	2 (4)	0
Via telephone	6 (12)	10 (20)	14 (29)	18 (37)	1 (2)
**Please select your estimate of the frequency with which pharmacists perform initial medication counseling through each of the following methods in most of your clinics: (select one for each method)**					
During a separate clinic visit in person	27 (55)	16 (33)	5 (10)	0	1 (2)
During provider visit in person	27 (55)	15 (31)	4 (8)	2 (4)	1 (2)
Via telemed video counseling	37 (76)	7 (14)	3 (6)	2 (4)	0
Via telephone	1 (2)	9 (18)	15 (31)	24 (49)	0

^a^All data are No. (%) of survey responses.

Compared to 2022, in 2024 there were higher rates of HSSPs reporting screening for necessary immunizations (98% vs 89%), counseling patients on recommended immunizations (89% vs 83%), and ordering immunizations through CPA (24% vs 16%), but fewer respondents reporting administering recommended immunizations (15% vs 23%).

### Treatment monitoring

For patients actively on treatment, HSSPs were asked 12 questions about treatment monitoring. Communication methods for patient monitoring were similar to those reported in 2022. All respondents use telephone communication, with 93% using it frequently or always. Comparatively, in-person communication and through the patient portal in the EHR were used only 16% and 23% frequently or always respectively. Novel communication options such as SMS messaging and telehealth video visits were both rarely used (76% and 84%, respectively) (Figure 11).

**Figure 11.**
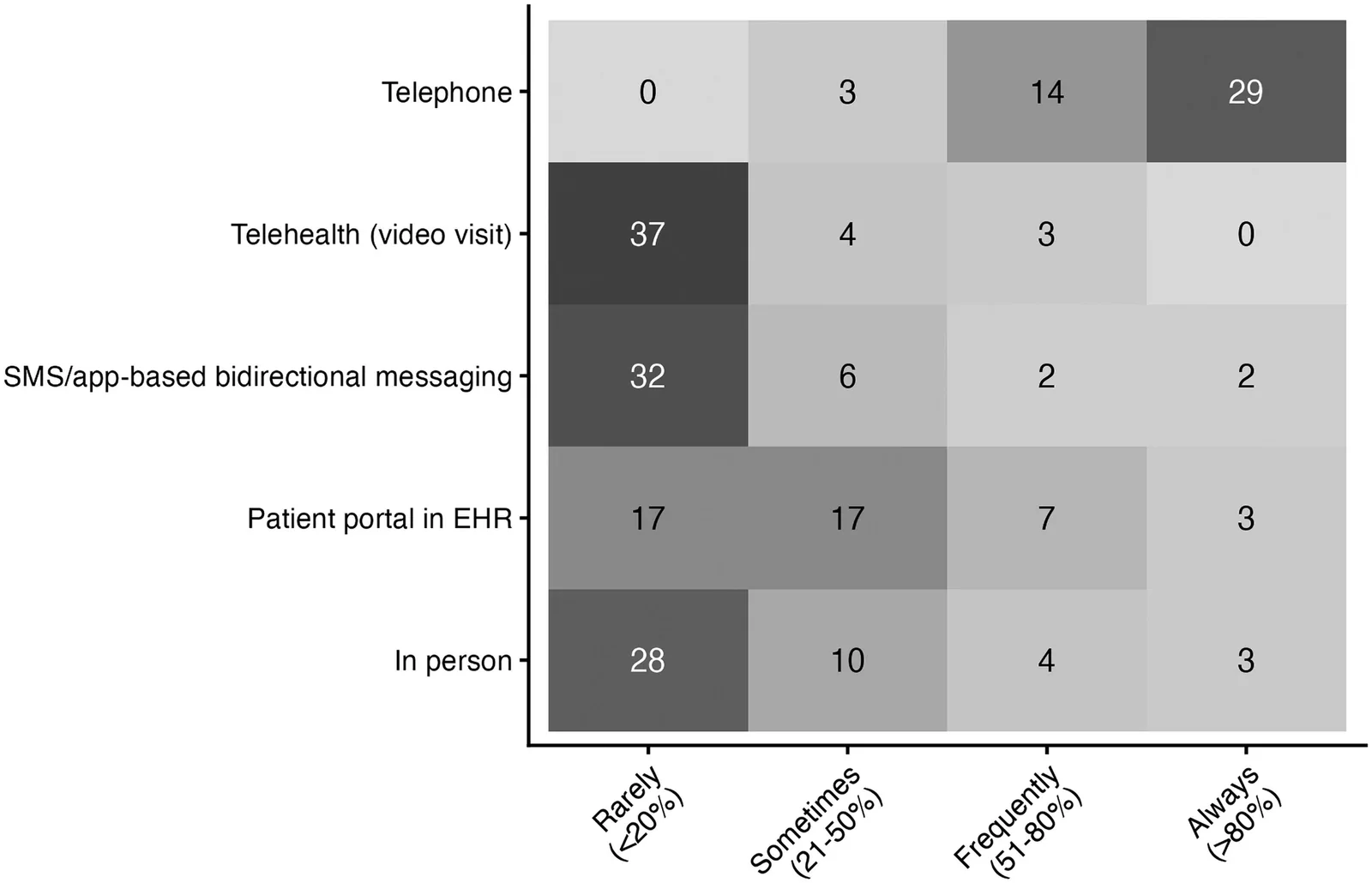
Health-system specialty pharmacies use various communication methods for patient monitoring. The numbers embedded in the cells represent raw counts of respondents to this question, with darker cells indicating higher counts.

For all interventions surveyed, the majority of HSSPs (>60%) reported both performing the interventions and documenting them in the EHR (Table 3). All HSSPs reported performing interventions related to adverse event management, coordinating specialty medication financial issues, discontinuing/recommending discontinuation of medication, identifying/mitigating drug-drug interactions, and providing adherence assessment or support. The least commonly performed interventions included ordering/recommending nonmedication supplies and performing immunization screening/administration (Table 3).

**Table 3.** Intervention Performance and Documentation by HSSP Staff (N = 47 Responses)^a^

Intervention	Not performed or documented	Performed and documented in EHR	Performed but not documented in EHR
Adverse event management	0	43 (91)	3 (6)
Coordinate nonfinancial specialty medication issues (eg, sites of care, discharge counseling, infusion coordination, clinic follow-up, social determinants of health)	4 (9)	31 (66)	11 (23)
Coordinate specialty medication financial issues	0	33 (70)	13 (28)
Discontinue/recommend discontinuation of medication	0	38 (81)	8 (17)
Identify/mitigate drug-drug interactions	0	40 (85)	6 (13)
Order/recommend ancillary drug initiation	4 (9)	35 (74)	7 (15)
Order/recommend dose adjustment	1 (2)	38 (81)	7 (15)
Order/recommend laboratory assessments/screenings	4 (9)	38 (81)	5 (11)
Order/recommend nonmedication supplies	7 (15)	30 (64)	9 (19)
Order/recommend specialty medication (new start or changes)	5 (11)	36 (77)	5 (11)
Perform immunization screening/administration	8 (17)	32 (68)	6 (13)
Provide adherence assessment or support	0	32 (68)	14 (30)

Abbreviations: EHR, electronic health record; HSSP, health-system specialty pharmacy.

^a^All data are No. (%) of survey responses.

Almost all HSSPs (94%) reported tracking patient response to therapy using disease state–specific outcomes, with 67% of respondents doing so in all or most patients or disease conditions. Similar to 2022, the most common reasons for reporting disease state–specific outcomes were accreditation requirements (87%), developing and executing quality improvement or research projects (76%), identifying patients who need intervention from the pharmacist (72%), and reporting to clinic providers (70%). Comparable to findings from 2022, most HSSPs (79%) reported that a single negative response or outcome triggered a pharmacist review or intervention, and 53% are able to respond based on a negative trend triggering an intervention or review.

The most-reported outcomes measures that were tracked and used in patient care were adverse events (77%), patient-reported goal progress (77%), relapse/flare/disease progression (75%), and quality of life (75%) (Figure 12). While most outcomes were tracked and used in patient care, time to medication fill (60%), patient satisfaction with HSSP (54%), adherence (via proportion of days covered [42%] or medication possession ratio [33%]) were most often tracked for reporting purposes only.

**Figure 12.**
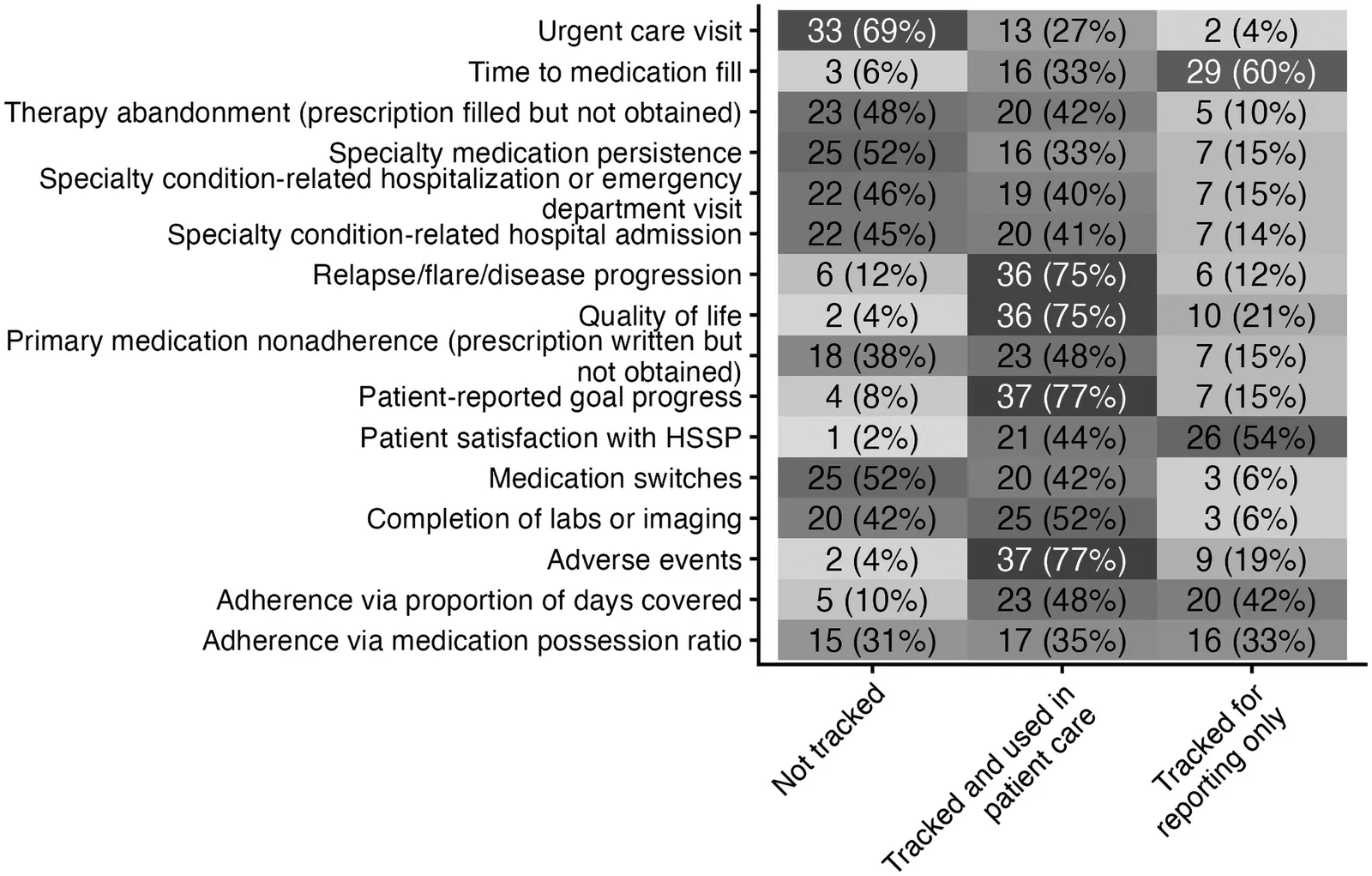
Health-system specialty pharmacies (HSSPs) track and use various outcomes metrics in patient care. A total of 49 respondents answered this question. The numbers embedded in the cells represent raw counts and percentages of respondents to this question, with darker cells indicating higher counts.

The frequency of patient assessments was most commonly decided by clinic-based standardized protocols (79%), medication factors such as adverse effects (75%), or patient or disease state factors (72%). The use of health-system standardized protocols decreased from 42% in 2022 to 21% in 2024. Among survey participants using patient or disease state factors to determine assessment frequency, the most common factors were duration on therapy (97%), adherence (88%) and patient response to therapy (a newly added response option in the 2024 survey) (85%), which was similar to results reported in 2022 (Figure 13). Medication factors that determined assessment frequency were commonly safety/effectiveness monitoring requirements (89%), anticipated adverse effect burden (80%), and medication complexity (newly added in 2024) (77%) (Figure 14).

**Figure 13.**
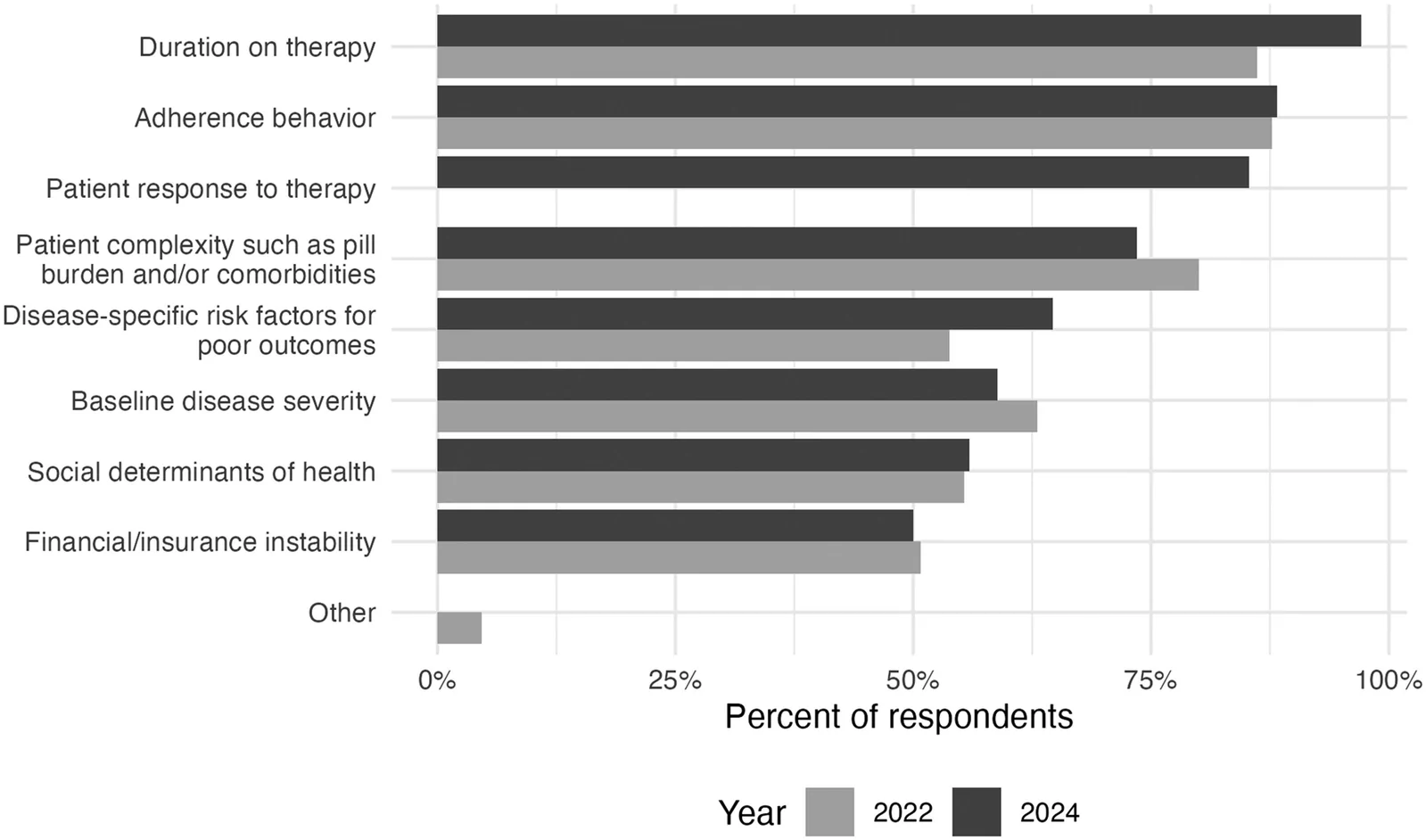
Patient or disease state factors used to determine the frequency of patient assessment.

**Figure 14.**
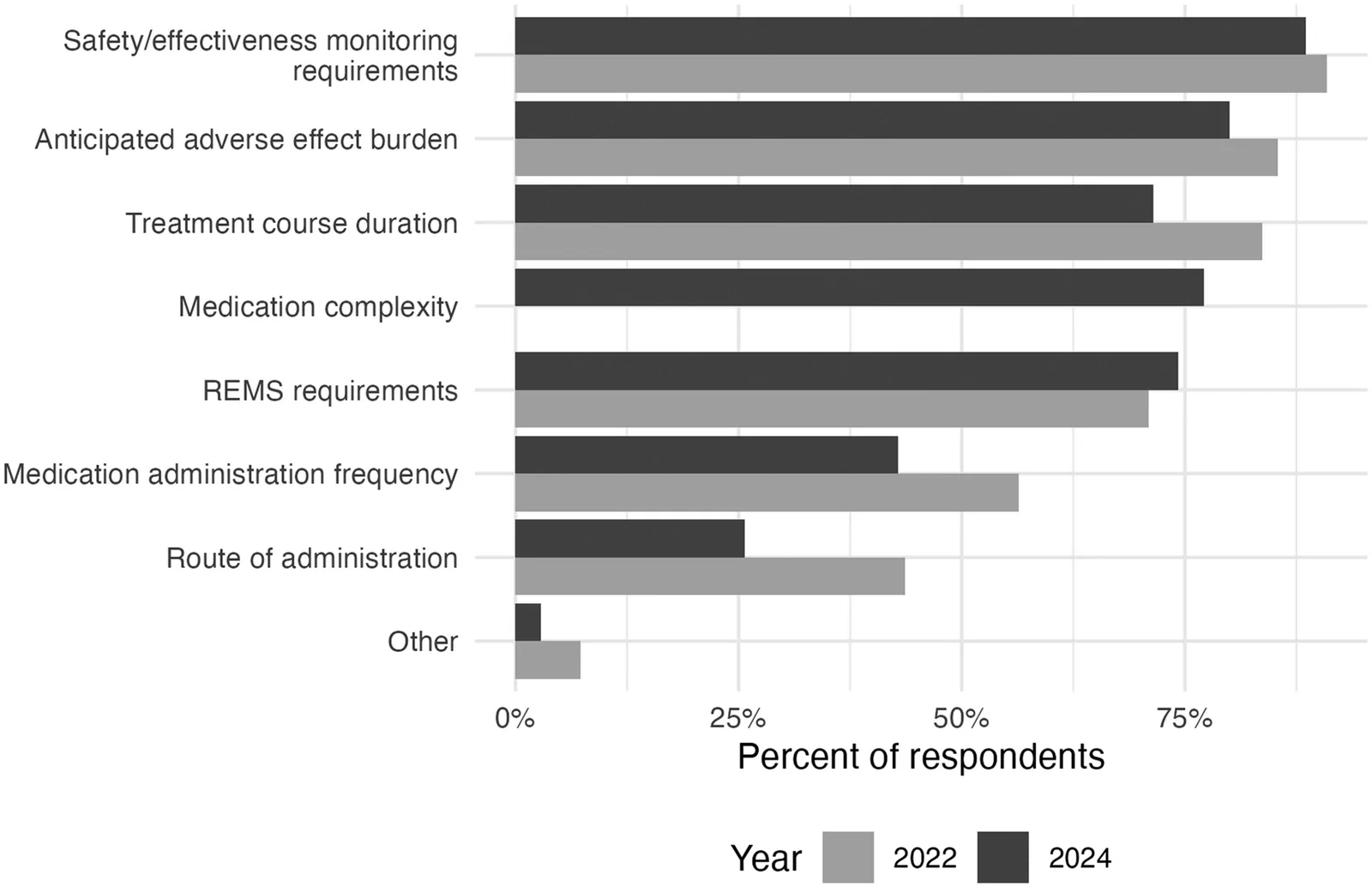
Medication factors used to determine the frequency of patient assessment. REMS indicates risk evaluation and mitigation strategies.

Overall, 94% of HSSPs reported at least one method of assigning risk categories to medications. Medication risk is primarily determined by medication complexity (62%), adverse effect severity or frequency (60%), medication designation such as REMS (60%), and importance of adherence to therapeutic outcomes (53%). When risk categories were assigned, they were utilized to determine assessment frequency (68%), patient/population monitoring (63%), and staff training (61%).

### Continuity of care services

There were 11 questions related to continuity of care services provided to specialty patients. Most respondents (77%) reported offering services beyond basic specialty medication prescription fulfillment, an increase from the 2022 results (69%) (Figure 15). Many HSSPs (64%) offer genetic counseling services. Immunization clinics were offered by 36% of respondents, and nonspecialty chronic disease management was offered by a third of respondents (34%).

**Figure 15.**
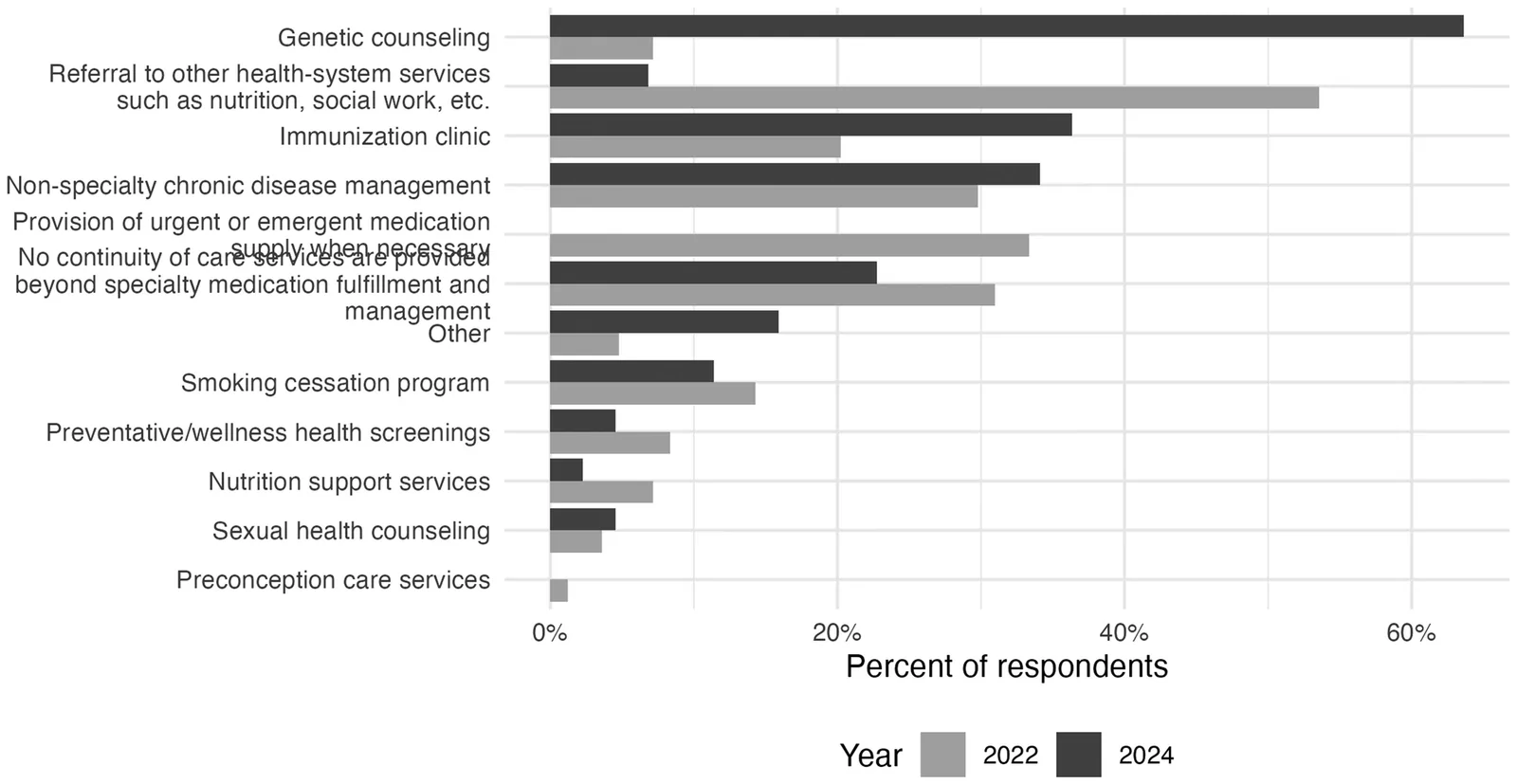
Services beyond specialty medication fulfillment and management offered by health-system specialty pharmacies (HSSPs). Most HSSPs (77.8%) offer some type of continuity-of-care service.

Waste avoidance practices were widespread among HSSPs, with most assessing patients’ on-hand medication before refills (96%), conducting clinical interventions to reduce medication and healthcare waste (91%), participating in treatment discontinuation or modification (87%), and reviewing specialty medications for clinical appropriateness prior to submitting PAs (76%). Over half of HSSP respondents (59%) were involved in some managed care activities, most commonly by collaborating with a health-system accountable care organization/payer groups to provide specialty medication management for non–health-system employees (36%). HSSP involvement in population health initiatives remained stable (54% in 2024 and 55% in 2022). Population health initiatives included collaborating with health-system population health services for patient care (37%), performing or referring patients for medication therapy management or comprehensive medication management services (30%), and developing or utilizing EHR screening tools for patient monitoring (30%).

For 43% of HSSPs that reported collecting and or using social determinants of health (SDOH) data, the information most commonly collected and/or used for patient care were related to financial resource strain (84%), depression (68%), and housing instability (68%) (Figure 16). Most HSSPs assess SDOH at treatment start (95%) and at pharmacist reassessment (74%). SDOH information was frequently obtained from the EHR using data previously collected by other healthcare professionals that is accessible through the EHR (90%) or via a SDOH assessment with the patient (63%). Thirty-seven percent of respondents reported that pharmacy technicians complete SDOH assessment with patients. HSSP patients burdened with negative SDOH are most commonly assisted by social workers (72%) and financial counselors/advocates (65%).

**Figure 16.**
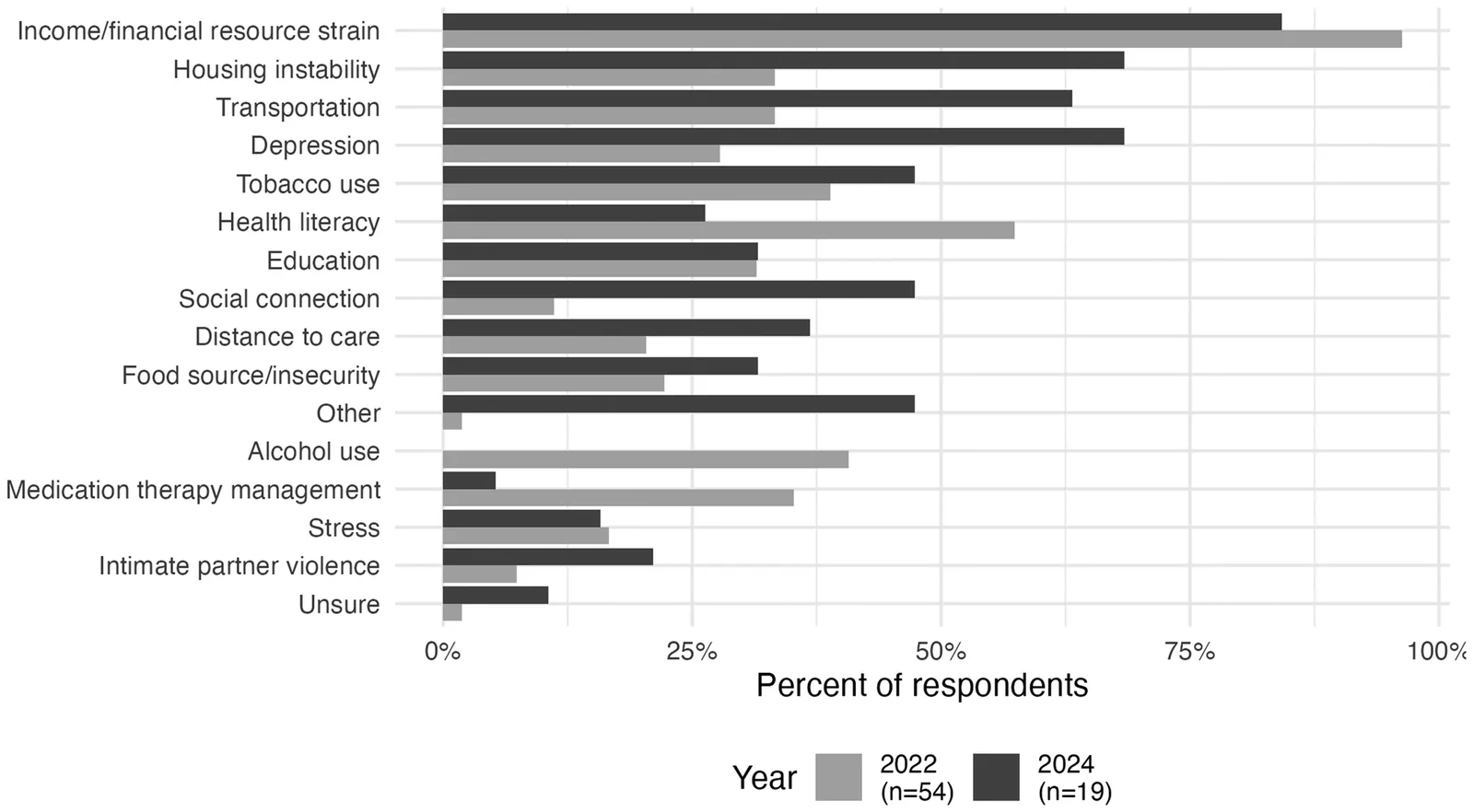
Social determinants of health variables collected and/or used by health-system specialty pharmacies for patient care.

HSSPs commonly coordinated care for specialty patients through multiple episodes of care across the continuum, such as insurance coverage/plan changes (80%) and navigating a patient through sites of care transitions (78%), different specialty or retail pharmacies (74%), different medication administration modalities (74%), and multiple healthcare prescribers (57%). The top 4 inpatient to outpatient transitions of care activities undertaken by HSSPs were assisting with medication procurement (72%), delivering medication to the home at the time of discharge (75%), delivering medications at the bedside and counseling the patient/caregiver prior to discharge (64%), and completing medication reconciliation for patients initiated on specialty medication while an inpatient (56%).

HSSPs reported using a wide range of technology to support patient engagement and integrative care (Figure 17). Among the types of technology currently used or planned to be implemented within 6 to 12 months for specialty pharmacy services, use of the health-system EHR patient portal to communicate with patients was most common (80%), followed by electronic refill requests (69%) (Figure 17). One-way and 2-way patient texting (both 47%), virtual visit/counseling (44%), secure emails (33%), and online chat (16%) were less common.

**Figure 17.**
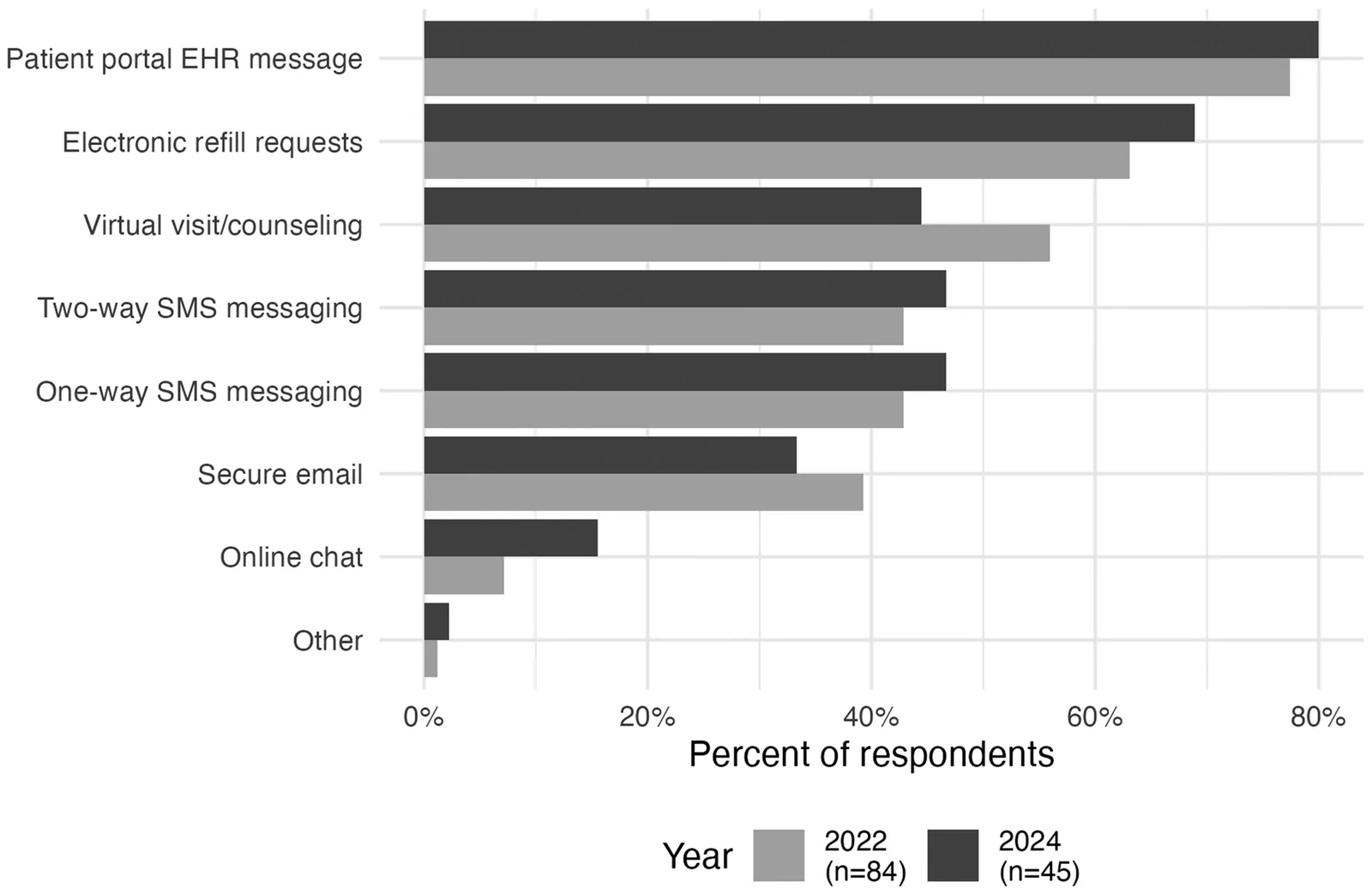
Current or planned use of technology by health-system specialty pharmacies for patient care. Most sites reported using electronic health record (EHR) patient portal messages and electronic refill requests.

### Education and training

There were 13 survey questions on education and training. About 40% of respondents reported providing an overview of specialty pharmacy services, programs, and medications to medicine learners or new providers, which decreased from 60% in 2022. The percentage of HSSPs that reported providing at least one educational session to clinic staff decreased from 80% in 2022 to 65% in 2024, with the most common education frequency being 3 to 4 times per year (28%). Specialty medication drug information was most commonly provided through clinic-embedded pharmacists (78%), in-service trainings (71%), and clinic staff meetings (62%). A total of 89% of respondents reported that their HSSP provided at least one continuing education (CE) presentation per year, which increased from 50% in 2022. Thirty-six percent of HSSPs reported providing 1 to 2 CE presentations, with fewer reporting 3 to 4 per year (20%), 5 to 10 per year (16%), or more than 10 per year (18%).

Approximately two-thirds of respondents reported producing at least one research publication each year, which increased from 45% in 2022. A total of 40% of respondents reported authoring 1 to 2 research publications each year, while fewer reported 3 to 4 (18%) or 5 to 10 each year (7%). At the time of the survey, 64% of HSSPs reported that at least one publication has been authored by their team to date, with 1 to 5 publications being the most common (31%), followed by 6 to 10 (2%), 11 to 15 (13%), 16 to 20 (7%), and more than 20 (11%) publications. Over half (51%) of respondents reported providing a research presentation each year, which decreased from 61% in 2022. When surveyed, 69% of HSSPs reported authoring at least one research presentation to date, with a total of 1 to 5 presentations being the most common (24%), followed by 6 to 10 (9%), 11 to 15 (7%), 16 to 20 (11%), and more than 20 (18%) presentations.

The most common ways HSSPs reported supporting outcomes research was through resident projects, clinical pharmacists without dedicated time provided, student projects, and national pharmacy organization involvement (Table 4). The percentage of respondents who reported support from clinical pharmacists without dedicated time increased from 45% in 2022 to 67% in 2024, and the percentage reporting support from resident projects and support from student projects also increased, from 74% to 80% and from 56% to 64%, respectively. The proportion of respondents who reported support for outcomes research through a clinical pharmacist with dedicated time decreased from 36% to 29%, and the proportion who reported having a dedicated outcomes pharmacist slightly increased, from 12% to 16%, from 2022 to 2024.

**Table 4.** Support for Outcomes Research^a^

Method	2022 (N = 85)	2024 (N = 46)
National pharmacy organization involvement	42 (49)	3 (7)
Resident projects	63 (74)	37 (80)
Clinical pharmacist with dedicated time provided	31 (36)	13 (28)
Student projects	48 (56)	30 (65)
Clinical pharmacist without dedicated time provided	38 (45)	31 (67)
Not supported/no initiatives	9 (11)	0
Dedicated outcomes pharmacist/FTE	10 (12)	7 (15)
Other	2 (2)	29 (63)
Outsourced (paid research agreements completed by nonpharmacy staff)	2 (2)	1 (2)

^a^All data are No. (%) of survey responses.

From 2022 to 2024, the percentage of HSSPs that reported having a longitudinal research experience for student pharmacists increased slightly from 24% to 29%. The experience was reported to be part of the school of pharmacy curriculum by nearly all respondents (92%). About half of respondents with a longitudinal research experience accepted 1 to 5 students (46%), followed by 5 to 10 (23%) and more than 10 (31%).

## Discussion

### Value of the advanced integrated practice model

The survey findings highlight that HSSPs are extensively integrated into the patient specialty medication journey and provide innovative, patient-focused services that improve access and outcomes, reduce waste, and drive practice advancement. Overall, HSSPs have become more deeply embedded in a broader range of clinics since the previous survey. Integration in these clinics enables collaborative conversations with providers around medication selection, safety screening, and patient response to therapy. CPA/CDTM arrangements deepen this integration as specialty pharmacists use these agreements to make therapeutic adjustments and order safety screenings. Additional research is needed to evaluate the operational and clinical impact of CPA/CDTM implementation in specialty clinics.

Most HSSPs reported providing patient services above and beyond prescription fulfillment. The number of HSSP-led immunization clinics nearly doubled from 2022 to 2024, which complements the prevalent role that HSSPs play in safety screening prior to initiation of specialty medications. HSSPs also more frequently reported providing pre-treatment counseling and injection training to patients compared to 2022. HSSPs have built on past successes by expanding into new clinical areas such as hemophilia, nephrology, and rare diseases.

The overall increase in HSSPs that report providing continuity of care services speaks to a broadened role for HSSPs within health systems. Patients treated for a specialty condition may be seen by several prescribers, including their primary care physician and other specialists.^8^ Their care may be further fragmented by payer-driven changes in therapy and in-network pharmacies. Healthcare policy changes such as the Inflation Reduction Act introduce new operational, financial, and supply chain implications to the specialty medication use process to which patients and healthcare providers alike will need to adapt. HSSPs consistently coordinate care through these common challenges, thereby establishing themselves as accessible providers who can help patients navigate the complex healthcare landscape.^9^

HSSPs continue to demonstrate the value of an advanced integrated practice model as programs mature beyond nascency. Most surveyed HSSPs are well established, and the maturation of these programs is generally reflected in growth in dispensing capacity and number of clinical areas serviced over time.

### Interprofessional optimization of EHR and transitions of care

The EHR continues to be a vital tool for HSSPs to access and document patient interactions and assessments, improving care team awareness and support for complex patient populations. Most interventions performed by HSSPs were reported as both performed and documented in the EHR, demonstrating the priority placed on transparency by HSSPs. Over 80% of respondents document adverse event management and orders/recommendations to discontinue medication, adjust doses, or order additional laboratory assessments or screenings. The importance of this transparency cannot be overstated. Providers are increasingly leaning on HSSPs prior to treatment selection, and HSSPs continue to build their systems to provide benefit and clinical assessment earlier in their processes, including through use of early treatment monitoring assessments.^10,11^

When prescriptions are sent to non-HSSP specialty pharmacies, providers lack insight into patient response to therapy and could waste valuable time to mitigate adverse events and keep patients adherent and persistent to therapy. HSSPs commonly reported performing and documenting interventions that can optimize specialty therapies and reduce waste, such as identifying and mitigating drug-drug interactions, recommending specialty medication discontinuation or dose adjustments, ordering/recommending ancillary treatments or laboratory assessments, and providing adherence support (Table 4). Rates of these interventions being performed and documented were similar to those reported in the 2022 survey. These results align with recent literature demonstrating the crucial interventions HSSP pharmacists provide. In patients initiated on oral anticancer medications, Phillips et al^11^ found that an early check-in protocol by HSSP pharmacists resulted in almost 1,700 interventions to address adverse effects and adherence, with 95.5% of pharmacist recommendations from those interventions being accepted. HSSP pharmacists practicing in a multiple sclerosis clinic avoided up to $156,265 of direct and indirect costs over just 6 months through pharmacist interventions.^12^ For patients with inflammatory bowel disease, one HSSP performed 2,141 clinical interventions over 1 year, most commonly to prevent lapses in therapy due to external pharmacy issues or insurance approval delays.^13^ Having these interventions communicated to the care team through the EHR elevates the care patients receive and the ability for providers to more quickly optimize treatment for best results.

Further enhancement of the EHR has been a commitment made by HSSPs, as demonstrated by the increase in technologies that these pharmacies currently offer or have planned. HSSPs are demonstrating value to their patients and providers through becoming “owners” of electronic prior authorization (ePA) processes housed within the EHR, as described by Choi and colleagues.^13^ Additionally, through EHR enhancement with multistep order transmittal (MSOT) Ngyuen et al^14^ found that HSSPs could significantly decrease the rate of represcribing by providers for the purpose of modifying the destination pharmacy. In the same study, the authors found that the EHR enhancement also led to a significant increase in represcribing associated with patient preference.^14^ Other research has shown that implementation of certain EHR capabilities can support standardization of workflow and increased transparency of the care provided by the HSSP team.^15^ Use of ePA and MSOT was not assessed in this survey, but that may be an area of interest for future surveys.

HSSPs continue to build their platforms to support discrete data collection. More HSSPs are tracking and using patient outcomes data, including relapse/flare/disease progression, quality of life, patient-reported goal progress, and adverse events. Summerlin and colleagues^16^ shared in an article a process used to develop and implement ambulatory pharmacy clinical outcomes measures, highlighting the importance of HSSPs continuing to invest in EHRs and increase the capture of discrete data elements related to patient outcomes. Similarly, O’Reilly et al^17^ developed predefined disease state goals for many clinical areas serviced by their HSSP and were able to meet 85% of the goals in the year evaluated, yielding $5,167,043 in direct cost savings. An increased number of HSSPs reported using disease-specific risk factors for poor outcomes to determine assessment frequency. Fitzpatrick et al^18^ described dashboards developed by their HSSP to standardize data capture and drive disease- and patient-specific interventions in support of delivering high-quality care. HSSPs are leveraging pharmacists’ clinical expertise, relationships with patients and providers, and access to patients’ clinical data to provide the highest level of care using standardized outcomes and innovative technologies. Development of these more expansive and individualized patient outcomes programs, coupled with the heightened level of other services offered by HSSPs, has been shown to improve clinical outcomes and lower healthcare costs.^19,20^ This level of integrated, data-driven care is an advanced capability not available in non-HSSP specialty pharmacies.

### Engagement with/expectations of payers and manufacturers

HSSPs are uniquely positioned to collect and provide robust and meaningful data. For payers and manufacturers that are currently partnering with HSSPs, the number and frequency of data elements reported continues to increase. While many capable HSSPs can meet the tracking and reporting imposed on them, the inability to meet reporting requirements is frequently cited by payers and manufacturers as the reason they are unable to provide an HSSP access to dispense for patients and/or medications. Though HSSPs provide a myriad of services to all patients of the health system being prescribed specialty medications, patients filling prescriptions with the HSSP typically receive more comprehensive clinical service support beyond medication access support. Continuing to improve HSSP contracting with payers and manufacturers is a priority area for many health systems, in order to improve the percentage of health system–generated prescriptions that patients are eligible to fill within the HSSP if patients want to use the HSSP.

As seen in this survey, HSSPs have expansive tracking and reporting capabilities. Not only can HSSPs meet what might be considered basic reporting demands, such as adherence and turnaround time statuses, but additional information is widely tracked and available. For example, the survey results indicated that for the majority of HSSPs, clinical interventions such as ordering or recommending dose adjustments are commonly both performed and documented in the EHR. Additionally, survey findings showed that more than three-quarters of HSSPs report on adverse events, patient-reported goal progress, and clinical outcomes such as relapse/flare/disease progression. Because an HSSP is integrated within a health system it has access to additional information in the EHR and can partner with the prescriber to both ensure patients therapy is safe and effective and intervene when it is not.

Despite numerous opportunities for reporting, most payers and manufacturers are not requesting this type of data. As HSSPs are uniquely positioned to provide this advantageous information, payers and manufacturers should partner with HSSPs to identify beneficial outcomes data and clearly define data needs so that HSSPs can provide the enhanced data that would also benefit payers and manufacturers.

Members of the HSSP community are actively engaged in efforts to develop standardized outcome and value metrics to demonstrate the unique value of HSSPs. The ultimate goal is to demonstrate to payers and manufacturers the improved value patients receive when utilizing an HSSP and the need for HSSPs to have access to serve more of the patients seen in their integrated clinics. The Outcomes and Value Section Advisory Group within the ASHP Section of Specialty Pharmacy Practitioners has developed standardized metrics dashboards for hepatitis C and multiple sclerosis, proposed a policy statement describing the need to include HSSPs in payer networks, and continues to work towards development of standardized clinical metrics.^21^ It will be imperative that HSSPs dedicate the necessary resources to support a strategy that allows HSSPs to proactively address defining reporting requirements from payers and manufacturers based on the enhanced reporting capabilities of HSSPs. Moreover, there is an ongoing opportunity for manufacturers and payers who collaborate with HSSPs to provide feedback on the results of the data that HSSPs report. Sharing standardized metric results and allowing the HSSP to evaluate their outcomes would create an opportunity to identify areas for improvement in patient care.

### Contributions to the specialty pharmacy body of literature

The results of this survey highlight the growing influence of HSSPs within the specialty pharmacy industry and highlight the willingness of HSSPs to advance practice through publication and presentation.

The volume and variety of HSSP publications and presentations have increased rapidly in recent years. Most HSSPs (72%) reported authoring at least one peer-reviewed research article to date. The ASHP Specialty Outcomes Literature Library is maintained by the Outcomes and Value Section Advisory Group within the ASHP Section of Specialty Pharmacy Practitioners and, at the time of writing, contained 179 publications related to specialty pharmacy practice or outcomes of patients prescribed specialty medications.^22^ These publications cover a variety of topics such as specialty pharmacy program development, efficiency, quality of care, financial outcomes, clinical/diagnosis-related outcomes, and adherence/persistence. Many publications in the outcomes library are from practitioners employed by HSSPs and include many examples demonstrating improved outcomes, reduced costs, and high-quality patient care within HSSP programs.

This survey indicates that many HSSPs publish or present research at least once annually, with most producing 1 to 10 research publications per year and some performing as many as 20 research presentations per year. This achievement is notable given the planning and resources required for research and publication. Although some HSSPs have dedicated outcomes research positions, most appear to rely on support from students, residents, and team members without research-specific positions.

HSSPs also reported a large increase in CE presentations. CE presentations play a critical role in maintaining up-to-date specialty pharmacy programs given how frequently new products, expanded indications, and updated guidelines become available. This strong engagement in developing and presenting CE reflects the commitment of HSSP team members to share their knowledge with their interprofessional colleagues and across the industry, serving as subject matter experts in specialty pharmacy practice.

### Strengths, limitations, and future directions

Strengths of this survey study include the targeted HSSP programs, with respondents representing a geographically diverse sample with a variety of years of maturation; the substantial volume of questions posed and the corresponding data describing current practices; and the timeliness of the survey, having been performed 2 years since the last survey. This survey was also notably shorter than the 2022 survey (64 questions vs 119 questions). Survey limitations include the smaller sample size compared to the 2022 survey, the potential for duplicate responses, and the survey length, which may have led to early termination by respondents. Additionally, because there is not a validated list of email addresses for HSSP staff and leaders, there was the potential for omitting HSSPs. The first question on the survey was an effective screener to ensure respondents were from HSSPs. Future surveys should explore the relationship between HSSP pharmacists and other department of pharmacy members such as ambulatory care pharmacists and inpatient pharmacists, as they often work in tandem and a better understanding of their roles would be useful.

## Conclusion

Though many clinical services and patient care offerings have remained consistent within HSSPs since the previous survey, this survey data demonstrates sustained progress in collaborating with providers, utilizing patient-specific outcomes, and leveraging data reporting to advance and optimize the specialty medication use process. These results describe how highly integrated HSSP pharmacists and pharmacy technicians are within clinic practice models and the unique value that HSSP clinical services bring to all patients prescribed specialty medications in a health system, which is further strengthened when patients are able to use the HSSP for prescription fulfillment. HSSPs have developed extensive treatment initiation strategies, monitoring strategies, and patient care resources that have contributed to optimizing patient outcomes.

## Data Availability

The data underlying this article are available in the article.
